# Celiac disease and primary hyperparathyroidism: an infrequent association

**DOI:** 10.31053/1853.0605.v80.n4.42137

**Published:** 2023-12-26

**Authors:** Luis Agustín Ramírez Stieben, Estefanía Pustilnik, Rodolfo Néstor Feldman, Diamela Bolzán, Iván Bedini

**Affiliations:** 1 Unidad de Tiroides y Paratiroides del Grupo Gamma (Rosario)

**Keywords:** enfermedad celíaca, hiperparatiroidismo primario, osteoporosis, celiac disease, primary hyperparathyroidism, osteoporosis, doença celíaca, hiperparatireoidismo primário, osteoporose

## Abstract

**Objective:**

Primary hyperparathyroidism (PHPT) and celiac disease (CD) are two distinct medical conditions that can affect bone health. While PHPT leads to excessive calcium levels and bone abnormalities, CD impairs calcium and vitamin D absorption due to small intestine damage.

**Case report:**

We present a case of a 49-year-old woman diagnosed with osteoporosis who was found to have both PHPT and CD. The patient underwent a successful minimally invasive parathyroidectomy, which resulted in decreased parathyroid hormone levels.

**Conclusion:**

This case highlights the rare coexistence of PHPT and CD and emphasizes the importance of considering secondary causes of osteoporosis in patients with low bone mass. Further studies are needed to explore the underlying mechanisms and potential links between PHPT and CD.

## Resumen

**Objetivos:**

El hiperparatiroidismo primario (HPTP) y la enfermedad celíaca (EC) son dos condiciones médicas distintas que pueden afectar la salud ósea. Mientras que el HPTP provoca niveles excesivos de calcio y anomalías óseas, la EC dificulta la absorción de calcio y vitamina D debido al daño en el intestino delgado.

**Caso clínico:**

Presentamos el caso de una mujer de 49 años diagnosticada con osteoporosis que también se le detectó tanto HPTP como EC. La paciente se sometió con éxito a una paratiroidectomía mínimamente invasiva, lo cual resultó en una disminución en los niveles de la hormona paratiroidea.

**Conclusión:**

Este caso resalta la rara coexistencia de HPTP y EC, y enfatiza la importancia de considerar causas secundarias de osteoporosis en pacientes con baja masa ósea. Se necesitan más estudios para explorar los mecanismos subyacentes y posibles vínculos entre HPTP y EC.

## Resumo

**Objetivos:**

O hiperparatireoidismo primário (HPTP) e a doença celíaca (DC) são duas condições médicas distintas que podem afetar a saúde óssea. Enquanto o HPTP leva a níveis excessivos de cálcio e anomalias ósseas, a DC prejudica a absorção de cálcio e vitamina D devido a danos no intestino delgado.

**Caso clínico:**

Apresentamos um caso de uma mulher de 49 anos diagnosticada com osteoporose que foi diagnosticada simultaneamente com HPTP e DC. A paciente foi submetida com sucesso a uma paratiroidectomia minimamente invasiva, o que resultou em redução nos níveis de hormônio paratireoidiano.

**Conclusão:**

Esse caso destaca a rara coexistência de HPTP e DC e enfatiza a importância de considerar causas secundárias de osteoporose em pacientes com baixa densidade óssea. Estudos adicionais são necessários para explorar os mecanismos subjacentes e possíveis conexões entre HPTP e DC.

**CONCEPTOS CLAVE**

Que se sabe sobre el tema.

El hiperparatiroidismo primario y la enfermedad celíaca pueden afectar la salud ósea causando osteoporosis y dificultades en la absorción de calcio y vitamina D.

Que aporta este trabajo.

Nuestro caso reporta una rara coexistencia de hiperparatiroidismo primario y enfermedad celíaca en una paciente con osteoporosis, destacando la importancia de considerar causas secundarias de osteoporosis en pacientes con baja densidad ósea.

**Divulgación**

El hiperparatiroidismo primario (HPTP) y la enfermedad celíaca (EC) son dos condiciones médicas que pueden afectar la salud ósea de manera diferente. El HPTP se caracteriza por niveles elevados de calcio en el cuerpo y puede causar problemas óseos, como osteoporosis. Por otro lado, la EC es una enfermedad autoinmune que daña el revestimiento del intestino delgado, dificultando absorción de calcio y vitamina y contribuyendo al desarrollo de osteoporosis. Esta asociación poco común destaca la importancia de considerar las causas subyacentes de la osteoporosis en pacientes con baja densidad ósea.

## Introduction

Primary hyperparathyroidism (PHPT) represents the most prevalent cause of hypercalcemia. PTHPT is diagnosed in the presence of hypercalcemia and an elevated or inappropriately normal parathormone (PTH) level. Skeletal symptoms can manifest as a combination of fragility fractures, skeletal deformities, and bone pain ^1^ . Celiac disease (CD) is an autoimmune disorder that primarily affects the small intestine and is triggered by the ingestion of gluten ^2^ . The classic symptoms of CD include malabsorption, chronic diarrhea, weight loss, and abdominal pain. CD can also manifest in various extra-intestinal complications, including osteoporosis, vitamin D deficiency, secondary hyperparathyroidism, and, less commonly, osteomalacia ^3, 4^ . The association between CD and PHPT is not fully defined ^5^ .

In this case, we present a patient who presents with osteoporosis and is diagnosed simultaneously with PHPT and CD.

## Case Report

A case is presented of a 49-year-old woman who presents for evaluation of osteoporosis. She had a personal history of migraines and had previously undergone surgeries, including cesarean sections and tonsillectomy. She denied tobacco and alcohol consumption and had no history of fractures. Her menarche occurred at the age of thirteen, and she had a hormonal intrauterine device in place. Her father had died from colorectal cancer, and she had second-degree relatives with inflammatory bowel disease and rheumatoid arthritis. Additionally, she reported occasional use of nonsteroidal analgesics and isotretinoin for acne treatment.

During the physical examination, it was noted that the patient had a weight of 54 kg and a height of 1.60 m. A regular heart rhythm was auscultated, and the abdomen appeared flat, soft, and depressible, with present bowel sounds and no evidence of organ enlargement. No palpable lesions were detected in the thyroid area. There were no peripheral edemas, melanotic skin hyperpigmentation, or jaundice observed in the conjunctiva or sclera.

The lumbar spine bone densitometry showed a T score of -1.8 and a Z score of -1.1, while the hip showed a T score of -3.0 and a Z score -2.5, and femoral neck bone showed a T score of -3.3 and Z score -2.6. Consequently, a laboratory evaluation was conducted, revealing a calcium level of 12.2 mg/dl, a phosphate level of 1.79 mg/d, a PTH level of 131 pg/ml, and positive autoimmunity for CD (Table 1).

**Table N° 1:** Biochemical characteristics of the patient

	Results	Normal range
Hematocrit (%)	37	34.9-44.5
Hemoglobin levels (g/dl)	11.8	12.5-15.5
Calcium level (mg/dl)	12.2	8.5-10.5
Phosphorus level (mg/dl)	1.79	2.5-4.5
Parathyroid hormone level (pg/ml)	131	15-65
25-OH vitamin D level (ng/ml)	36	>30
24-hour urinary calcium (mg/day)	385	100-250
24-hour urinary phosphate (mg/day)	1086	400-1300
Tubular reabsorption of phosphate (%)	58	85-95.5
Beta Crosslaps (ng/ml)	0.94	0.131-0.670
Alkaline phosphatase (IU/l)	77	35-105
Creatinine (mg/dl)	0.76	0.5-0.9
Thyroid-stimulating hormone level (mIU/l)	2.58	0.27-4.2
Electrophoretic proteinogram	Normal	Normal
Immunoglobulin A (mg/dl)	275	70-400
Anti-transglutaminase IgG antibodies (IU/ml)	133	<10
Anti-transglutaminase IgA antibodies (IU/ml)	200	<10
Urinary free cortisol (µg/day)	49.4	36-137
Follicle-stimulating hormone (IU/l)	9.4	2.5-10.2
Estradiol (pg/ml)	343	40-398

The ultrasound of the parathyroid glands revealed a solid nodule measuring 24 x 13 mm, hypoechoic, and located adjacent to the lower pole of the right lobe (Figure 1A and Figure 1B). 99mTc-sestamibi scintigraphy with SPECT showed a focal area of intense radiotracer uptake, below the right thyroid lobe, posterior to the trachea, and anterior to the vertebral column (Figure 1C and Figure 1D).

**Figure N° 1.**
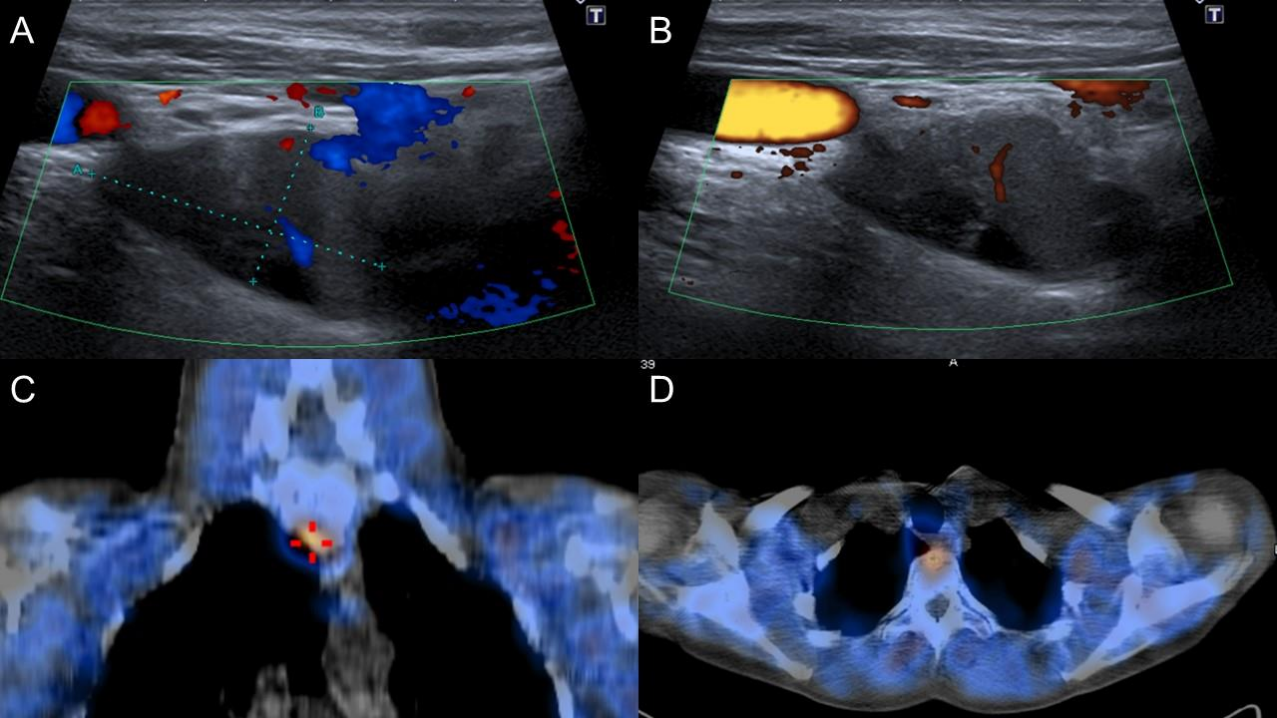
Preoperative parathyroid localization studies. A and B. Parathyroid ultrasound (sagittal orientation). Adjacent to the lower pole of the right lobe, a mixed, solid formation of approximately 24 x 13 mm is observed, which shows central flow on color Doppler. C and D. 99mTc-sestamibi scintigraphy with SPECT [coronal (C) and axial orientation (D)]. Focus of intense tracer-uptake, located below the right thyroid lobe, behind the trachea, and in front of the spine.

An upper gastrointestinal endoscopy was performed, which revealed low-height, comb-like Kerckring folds in the duodenum. The histopathological examination showed moderate to severe chronic duodenitis associated with marked and diffuse villous flattening and crypt hyperplasia. The estimated count of intraepithelial lymphocytes was forty elements per 100 enterocytes, consistent with a type IIIC lesion of the Marsh classification. These findings indicated severe damage to the small intestinal mucosa, suggestive of CD.

A minimally invasive parathyroidectomy (PTX) was performed. The lower right parathyroid gland was located and successfully removed. Intraoperative PTH levels significantly decreased following the resection of the lesion. The histopathological examination revealed a 35 mm nodular lesion without signs of atypia, capsular invasion, lymphovascular invasion, or perineural involvement, consistent with a parathyroid adenoma.

## Discussion

Osteoporosis is a prevalent condition characterized by reduced bone strength, leading to an increased risk of fractures ^6^ . Managing patients with confirmed osteoporosis or low bone mass (osteopenia) involves assessing fracture risk, investigating potential secondary causes of skeletal fragility, deciding on treatment initiation, and considering relevant clinical factors that may impact patient management. Secondary osteoporosis refers to osteoporosis caused by factors other than estrogen deficiency or aging. It has been suggested by some experts that a Z-score below the expected range for age and sex (e.g., below -2.0) may indicate a higher likelihood of secondary osteoporosis ^7^ . However, since secondary osteoporosis is common, it is more effective to screen all patients with osteoporosis for potential underlying causes ^8^ . In our case, the presence of a Z-score of -2.6 in the femoral neck and a Z-score of -2.5 in total hip raised suspicions about the presence of secondary osteoporosis. After further evaluation, the patient was diagnosed with PTHP and CD.

PHPT is the most common cause of hypercalcemia in the outpatient setting^1^. Epidemiological studies and prospective cohort data have provided evidence of an increased risk of both vertebral and non-vertebral fractures ^9^ . Post PTX, improvements in the microarchitecture, geometry, cortical thickness, and estimated bone strength translate into a significant decrease in the incidence of fractures ^10^ . On the other hand, CD can lead to malabsorption of calcium and vitamin D due to villous atrophy and inflammation in the small intestine, impairing their absorption. Approximately 25% of patients may develop secondary hyperparathyroidism, which is characterized by elevated PTH levels and serum calcium levels in the normal to low range, along with increased bone turnover ^11^ . A study of 103 patients found that 21% had low vitamin D levels, and alkaline phosphatase, an indicator of osteomalacia, was elevated in approximately 10% of patients ^12^ . In our patient, we did not demonstrate vitamin D deficiency or biochemical findings consistent with osteomalacia.

Although secondary hyperparathyroidism is common in patients with CD ^11^ , the association between HPTP and CD appears to be coincidental, as only a limited number of case reports have been published on this topic ^5,13, 14^ . In a single-center observational study, Maida et al. reported a prevalence of 2.3% for HPTP in patients with CD ^5^ . This is significantly higher than the reported prevalence of 2-3 in 1000 in the general population cited in most studies^1^. A large population-based study found a 2-fold increased risk of PHPT (42/100000 person-years) in patients with CD respect to nonceliac control group (22/100000 person-years) ^15^ . However, the authors do not rule out the possibility that part of the increased risk of PHPT may be due to biases, as the highest risk estimates were found in the first year after diagnosis when surveillance of patients with CD is more intense. However, an alternative explanation is that treatment for CD may have "unmasked" PHPT, and that the treatment of CD allows for improved absorption of calcium and vitamin D, resulting in the emergence of hypercalcemia and subsequent diagnosis of PHPT. Finally, long-standing undiagnosed CD could lead to parathyroid hyperplasia and tertiary hyperparathyroidism. Although the Ludvigsson cohort did not specifically report the frequency of four-gland hyperplasia ^15^ , the small case series by Maida et al. ^5^ reported that parathyroid adenoma, rather than hyperplasia, was the cause of the disease in all of their patients, as was the case with our patient.

## Conclusion

This case highlights the uncommon association between PHPT and CD. The coexistence of both conditions has diagnostic and therapeutic implications as they affect bone health. It is important to consider secondary causes of osteoporosis in patients with low bone density. Further research is needed to understand the underlying mechanisms and potential links between PHPT and CD.
